# Knowledge, Awareness, and Interest in Forensic Odontology Among the Dental Teaching Staffs in the Dental Colleges of Jharkhand

**DOI:** 10.7759/cureus.41884

**Published:** 2023-07-14

**Authors:** Ankur Bhargava, Sonal Saigal, Ranjeet Kumar, Silpi Chatterjee, Irfanul Haque, Dushyantsinh Vala

**Affiliations:** 1 Oral Pathology and Microbiolgy, Hazaribag College of Dental Sciences and Hospital, Hazaribag, IND; 2 Oral Pathology, Microbiology and Forensic Odontology, Rajendra Institute of Medical Sciences, Ranchi, IND; 3 Oral and Maxillofacial Surgery, Hazaribag College of Dental Sciences and Hospital, Hazaribag, IND; 4 Public Health Dentistry, Dr. D. Y. Patil Dental College & Hospital, Pune, IND; 5 Orthodontic and Dentofacial Orthopaedics, Hazaribag College of Dental Sciences and Hospital, Hazaribag, IND; 6 Oral and Maxillofacial Pathology and Oral Microbiology, Government Dental College & Hospital, Jamnagar, IND

**Keywords:** dental teaching staff, forensic odontology, interest, awareness, knowledge

## Abstract

Background: Forensic dentistry is a subspecialty of forensic science that handles, examines, and presents evidence from teeth in the most ethical manner possible. In addition to doing research, forensic odontology (FO) entails managing, examining, assessing, and presenting evidence from dentistry in civil or criminal investigations. In these circumstances, the forensic odontologist aids the court system by reviewing the dental findings.

Aim: The present investigation was carried out to assess the dental faculty member's awareness, interest, and knowledge of FO at dental educational institutions in Jharkhand, India.

Methods and materials: The sample size calculation assumes a simple random sampling technique and a large population size using the formula E=sqrt((Z^2*p*(1-p))/n). A total of 102 dental teaching faculty members from diverse dental specialties participated in the survey. Utilizing a validated questionnaire that was sent directly after receiving approval from the institutional ethics committee in January 2023, data were gathered in a tailored manner. The questionnaire included 12 questions to gauge the dental teaching faculty members' awareness, knowledge, and interest in FO. Closed-ended questions were included. The outcomes were computed using a percentage system.

Results: In this study, 95% of study participants agreed with the fact that teeth are a possible source of DNA, while 5% were unaware of this fact. 68% of study participants said that visual examination constitutes the initial stage in the identification process for unidentified bodies while 8% of study participants said that DNA fingerprinting constitutes the initial stage in the identification process for unidentified bodies. However, 8% of study participants didn’t know anything about this aspect. 72% of dental teaching faculty members agreed to the fact that Barr bodies should be used to determine sex. 89% of study participants responded positively to the question "Can teeth or enamel serve as a tool for determining age?" 11% of study participants did not know that teeth or enamel can serve as a tool for determining age. 41% of study participants had the correct information that FO instruction for bachelor of dental surgery (BDS) students takes place and, as per Dental Council of India (DCI) standards, should be given in both BDS second and third years.

Conclusion: The results of the current survey provided information on FO practices among Jharkhand dental institutes' dental faculty. The poll revealed that they have the necessary information, which they must have learned either while studying, participating in continuing dental education, or teaching.

## Introduction

Recognition of the person who was assaulted or the method of the offense is a process of brainstorming in the current situation when instances of crime are at their highest. Through the specialty of forensic odontology (FO), the dentist plays a small but crucial role in criminal investigations. In accordance with Keiser-Nielson, forensic dentistry is a subspecialty of forensic science that handles, examines, and presents evidence from teeth in the most ethical manner possible [1]. In addition to doing research, FO entails managing, examining, assessing, and presenting evidence from dentistry in civil or criminal investigations. In these circumstances, the forensic odontologist aids the court system by reviewing the dental findings [2].

Even though forensic dentistry has made great strides in terms of technical development on an international level, India continues to lag far behind other countries in this area. The necessity for dental professionals to be well-versed in FO is growing, as it is important for identifying people and spotting abuse in people at every stage of life. Dental professionals are medical specialists who regularly examine patients' head and neck regions while having a great possibility of spotting neglect and assault symptoms [3,4]. Every dentist needs to be aware of the forensic consequences of their work. In India, dental professionals are less frequently engaged in forensic situations due to a lack of proper awareness, expertise, and exposure, among others [5,6].

Although numerous studies have been done on the awareness, interest, and knowledge of general dental practitioners who are affiliated with dental educational institutions and are regularly exposed to academia, the most recent news, documents, and so on, their understanding is regularly updated; however, it is questionable as to how much interest they have in forensics [7,8]. So, the current investigation was conducted to evaluate the awareness, interest, and knowledge of FO among dental faculty members at dental educational institutions in Jharkhand, India.

## Materials and methods

Different dental colleges in Jharkhand, India, undertook the current cross-sectional survey. Ethical clearance was obtained from Hazaribag College of Dental Sciences and Hospital with the institutional reference number HCDSHIEC/2022/32.

The sample size calculation formula is as follows: E=sqrt((Z^2*p*(1-p))/n), where, Z=Z-value for the desired confidence level, p=estimated proportion or expected prevalence, and E=margin of error. Substituting the values: E=sqrt((1.96^2*0.5*(1-0.5))/00) E=0.098

So, with a desired sample size of 100 patients, and assuming a 95% confidence level, the margin of error would be approximately 9.8%. This sample size calculation assumes a simple random sampling technique and a large population size.

A total of 102 dental teaching faculty members from diverse dental specialties participated in the survey. The study included those dental students who were willing and available to participate and excluded all faculty, staff, and dental students not willing to participate in the study. The participant was made to seat in the outpatient room of the hospital. Utilizing a validated questionnaire that was sent directly after receiving approval from the institutional ethics committee in January 2023, data were gathered in a personalized manner. The questionnaire included 12 questions to gauge the dental teaching faculty members' awareness, knowledge, and interest in FO as shown in Table 1.

**Table 1:** Questionnaire for survey BDS, bachelor of dental surgery; DCI, Dental Council of India; FO, forensic odontology

Questions regarding knowledge
Are teeth a possible source of DNA?
What constitutes the initial stage in the identification process for unidentified bodies?
Can the Barr bodies be used to determine sex?
Can tooth/enamel serve as a tool for determining age?
Which year does FO instruction for BDS students take place, as per DCI standards?
Questions regarding awareness
What might you do when you found evidence of child abuse?
Are there any forensic odontologists in India, do you know?
Do you have any knowledge of a criminal proceeding that was resolved with the use of forensic dentistry?
Where did you learn about your expertise in forensic dentistry?
Questions regarding interest among dental teaching faculty members
Can we, as dentists, assist forensic experts by keeping records?
Do you know of any accredited FO educational institutions in India?
Would you choose to go through any such learning, if given the chance?

The knowledge-related questions aimed to evaluate the participants' understanding of fundamental concepts and principles of FO. The awareness-related questions aimed to assess the participants' familiarity with current practices, advancements, and real-world applications of FO. The interest-related questions aimed to gauge the participants' inclination toward incorporating FO into their teaching practices and professional development.

The Cronbach's alpha value for the questionnaire was 0.8, and the face value was 1. Closed-ended questions were included. The outcomes were computed using a percentage system. The collected data were analyzed using IBM SPSS Statistics, Version 22.0 (Armonk, NY, USA). Data were analyzed using the Chi-square test.

## Results

In this study, 95% of study participants agreed to the fact that teeth are a possible source of DNA, while 5% were unaware of this fact. 68% of study participants said that visual examination constitutes the initial stage in the identification process for unidentified bodies. 8% of study participants said that DNA fingerprinting constitutes the initial stage in the identification process for unidentified bodies. However, 24% of study participants didn’t know anything about this aspect. 72% of dental teaching faculty members agreed to the fact that Barr bodies should be used to determine sex. 13% of study participants didn’t agree with the fact that Barr bodies should be used to determine sex. 15% of study participants didn’t know about the fact that Barr bodies can be used to determine sex. 89% of study participants responded positively to the question that a tooth or enamel can serve as a tool for determining age. 11% of study participants didn’t know that teeth or enamel can serve as a tool for determining age. 41% of study participants had the correct information that FO instruction for bachelor of dental surgery (BDS) students takes place and, as per Dental Council of India (DCI) standards, should be given in both BDS second and third years. 29% of study participants replied BDS final year, 16% replied BDS third year, and 14% replied BDS second year (Table 2).

**Table 2:** Data regarding knowledge of FO BDS, bachelor of dental surgery; DCI, Dental Council of India; FO, forensic odontology

Are teeth a possible source of DNA?				
Agree		Disagree		Don’t know
95%		-		5%
What constitutes the initial stage in the identification process for unidentified bodies?				
Fingerprinting	Visual examination	Serological and genetic DNA comparison	Don’t know	Physical and anthropological examination
8%	68%	-	24%	-
Can the Barr bodies be used to determine sex?				
Agree		Disagree		Don’t know
72%		13%		15%
Can tooth/enamel serve as a tool for determining age?				
Agree		Disagree		Don’t know
89%				11%
Which year does FO instruction for BDS students take place, as per DCI standards?				
BDS second year	BDS third year	BDS Final year		Both a & b
14%	16%	29%		41%

41% of study participants, when asked "What might you do when you find evidence of child abuse?" replied they would inform the parents of the children. 100% of study participants, when asked "What might you do when you find evidence of child abuse?" replied they would inform the police while 18% of study participants when asked "What you might do when you find evidence of child abuse?" replied correctly that they would inform NGOs. 59% of study participants, when asked "Are there any forensic odontologists in India you know of?" replied yes, while 41% of study participants replied no. 56% of study participants replied yes when asked "Do you have any knowledge of a criminal proceeding that was resolved with the use of forensic dentistry?." 44% of study participants replied no when asked this question. 92% of study participants said that radio, mobile, electronic sources (e.g., TV), and so on are sources from which they learned about expertise in forensic dentistry. 8% of study participants said that seminars, journals, lectures, and others are sources from which they learned about expertise in forensic dentistry (Table 3).

**Table 3:** Data showing awareness regarding FO FO, forensic odontology

What might you do when you find evidence of child abuse?			
Inform police	Inform NGOs	Inform parents	Take no action
100%	18%	41%	
Are there any forensic odontologists in India you know of?			
Yes		No	
59%		41%	
Do you have any knowledge of a criminal proceeding that was resolved with the use of forensic dentistry?			
Yes		No	
56%		44%	
Where did you learn about your expertise in forensic dentistry?			
Radio, mobiles, electronic sources (e.g., TV)		Seminars, journals, lectures, etc.	
92%		8%	

**Figure 1:**
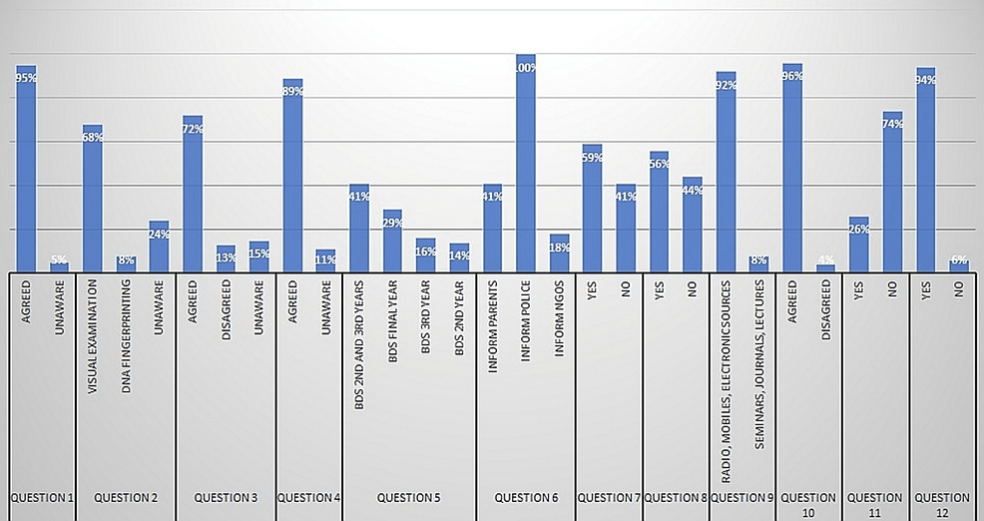
Summary of the results in graphical form

96% of study participants agreed to the fact that we can, as dentists, assist forensic experts by keeping records while 4% of study participants disagreed. 26% of study participants replied yes when asked "Do you know of any accredited FO educational institutions in India?." 74% replied no. 94% of study participants replied yes when asked "Would you choose to go through any such learning if given the chance?" while 6% of study participants replied no (Table 4, Figure 1).

**Table 4:** Interest among the dental faculty members FO, forensic odontology

Can we, as dentists, assist forensic experts by keeping records?		
Agree	Disagree	Don’t know
96%	4%	-
Do you know of any accredited FO educational institutions in India?		
Yes	No	
26%	74%	
Would you choose to go through any such learning, if given the chance?		
Yes	No	
94%	6%	

## Discussion

In a number of prosperous countries around the world, the practice of forensic dentistry has grown in importance. But it has not quite taken off in developing nations like India [9,10]. In the expanding realm of medicine, dental professionals would need to be more knowledgeable and cognizant of FO [11,12]. The present investigation was carried out to assess the dental faculty members at dental educational institutions in Jharkhand and India's awareness, interest, and knowledge of FO.

One of the most difficult topics in the study of FO is human identification. The simplest and most basic stage in identifying the unclaimed person is still a visual inspection. In the current study, 68% of participants favored it. For personal, societal, and legal reasons, it is necessary to identify a person [13,14]. As a result, one of the key goals of FO is person identification. Body identification by buccal smears is one of the most straightforward methods for determining gender, despite the fact that dimorphism in dogs is a fairly common method [15]. The inactive X chromatin known as the Barr body is seen in females against the nuclear membrane. These are lacking in males who are believed to have negative chromatin, while they exist in 40% of females who are thought to have positive chromatin. The pulp of human teeth in dead bodies can be used to determine a person's sex for up to four weeks [16]. Despite the fact that numerous studies have been conducted on the awareness, interest, and knowledge of general dental practitioners who are associated with dental educational institutions and regularly exposed to academia, the most recent news, and documents, among others, their interest in forensics is debatable [17].

Child abuse is a severe societal issue with global implications that is spreading alarmingly across all socioeconomic tiers and all racial or ethnic communities. All instances of child abuse, which are increasingly prevalent in daily life, should be discovered as soon as possible. Child abuse and neglect should be disclosed to childcare officials as soon as they are discovered because they result from a dearth of communication between the caretaker and the child and cause nonaccidental injury to the child's physical and developmental status [18]. According to research, parents may physically assault their children, and roughly one in ten children encounters this abuse.

The general public's level of awareness is significantly influenced by the media. The same is true for the current poll, where 92% of respondents indicated that TV series, radio, the internet, and other sources were their primary sources of information. Journals and publications are still among the most reliable and accurate sources of this information. The current analysis reveals that very few dental professionals keep up with forensic-related journals or publications [19].

In the present, when crime rates are at their peak, identifying the victim or the manner of the assault requires creative problem-solving. The dentist plays a small but important role in criminal investigations thanks to the field of FO. A branch of forensic science known as "forensic dentistry" handles, evaluates, and presents dental evidence in the most morally upstanding way possible. Most researchers in anthropology, archaeological researchers, and forensic odontologists use teeth as a credible method for estimating age-related alterations to teeth [20].

The study has a few limitations, such as a small sample size and the fact that only dental faculty members participated in it. For more accurate results, participants from other fraternities should be included as well. There are not many FO workshops or conferences held annually for dental surgeons, which may awaken students' interest in learning more about the field. The data collected relied on self-reporting by the participants. This introduces the possibility of recall bias or participants providing socially desirable responses, which may not accurately reflect their actual knowledge or practices. The study participants were selected from a specific group of dental teaching faculty members, which could introduce bias. It is possible that those who agreed to participate in the study had a higher interest or awareness in FO compared to non-participants. The study utilized a cross-sectional design, which captures data at a single point in time. This limits the ability to establish causality or assess changes over time. The study did not account for potential confounding variables that could influence the results. Factors such as participants' years of teaching experience, educational background, or exposure to FO training may have influenced their responses.

Conducting studies with larger sample sizes and including dental faculty members from different regions and countries would enhance the generalizability of the findings. The study exhibits both self-reporting bias and selection bias. These biases can impact the generalizability and reliability of the study findings. Longitudinal designs that follow participants over time can provide insights into changes in awareness, knowledge, and practices of dental teaching faculty members and identify factors that influence these changes. Further research with more diverse samples is needed to enhance the generalizability of the results to a broader population of dental teaching faculty members.

## Conclusions

The results of the current survey provided information on forensic odontology practices among Jharkhand dental institutes' dental faculty. The poll revealed that they have the necessary information, which they must have learned either while studying, participating in continuing dental education, or teaching. The survey also analyzed the associated variables of awareness and interest, which were found to be lower among dental teaching staff.

If held for dental surgeons on a regular basis, conferences, workshops, CDEs, and seminars will enhance their knowledge and sensibility. More dentists will be interested in attending and taking part in such programs if additional career possibilities in this industry are created.
